# Don’t mind if I do: Arctic humpback whales respond to winter foraging opportunities before migration

**DOI:** 10.1098/rsos.230069

**Published:** 2023-09-06

**Authors:** Lisa Elena Kettemer, Theresia Ramm, Fredrik Broms, Martin Biuw, Marie-Anne Blanchet, Sophie Bourgeon, Paul Dubourg, Anna C. J. Ellendersen, Mathilde Horaud, Joanna Kershaw, Patrick J. O. Miller, Nils Øien, Logan J. Pallin, Audun H. Rikardsen

**Affiliations:** ^1^ UiT—The Arctic University of Norway, Faculty of Bioscience, Fisheries and Economics, 9037 Tromsø, Norway; ^2^ North Norwegian Humpback Whale Catalogue (NNHWC), Straumsvegen 238, 9109 Kvaløya, Norway; ^3^ IMR Institute of Marine Research, FRAM—High North Research Centre for Climate and the Environment, 9007 Tromsø, Norway; ^4^ Norwegian Polar Institute, FRAM—High North Research Centre for Climate and the Environment, 9007 Tromsø, Norway; ^5^ Norwegian Institute for Nature Research, FRAM—High North Research Centre for Climate and the Environment, 9007 Tromsø, Norway; ^6^ Sea Mammal Research Unit, Scottish Oceans Institute, University of St Andrews, KY16 9ST St Andrews, UK; ^7^ IMR Institute of Marine Research, Nordnes, PO Box 1870, 5817 Bergen, Norway; ^8^ Department of Ecology and Evolutionary Biology, UC Santa Cruz, Santa Cruz, CA 95060, USA

**Keywords:** marine mammals, migration timing, stopover, spatial ecology, hormone profiling, pregnancy rates

## Abstract

Migration patterns are fundamentally linked to the spatio-temporal distributions of prey. How migrating animals can respond to changes in their prey's distribution and abundance remains largely unclear. During the last decade, humpback whales (*Megaptera novaeangliae*) used specific winter foraging sites in fjords of northern Norway, outside of their main summer foraging season, to feed on herring that started overwintering in the area. We used photographic matching to show that whales sighted during summer in the Barents Sea foraged in northern Norway from late October to February, staying up to three months and showing high inter-annual return rates (up to 82%). The number of identified whales in northern Norway totalled 866 individuals by 2019. Genetic sexing and hormone profiling in both areas demonstrate a female bias in northern Norway and suggest higher proportions of pregnancy in northern Norway. This may indicate that the fjord-based winter feeding is important for pregnant females before migration. Our results suggest that humpback whales can respond to foraging opportunities along their migration pathways, in some cases by continuing their feeding season well into winter. This provides an important reminder to implement dynamic ecosystem management that can account for changes in the spatio-temporal distribution of migrating marine mammals.

## 1. Introduction

The spatio-temporal distribution of prey resources is considered foundational to animal movement. Seasonal migrants, in particular, rely on predictably occurring resources to fuel their year-round energy demands [1,2]. However, ecosystems undergo changes at multiple scales as a result of natural variability, anthropogenic drivers, or a combination of these. For example, cyclical changes can occur naturally on decadal scales or interannually (e.g. fluctuations in ice cover and prey distributions), and anthropogenically caused alterations can be sudden (e.g. construction projects), or gradual (e.g. climate change or pollution). Such variability in the physical environment can cause changes that cascade through the food web, resulting in shifts in the timing and spatial distribution of prey aggregations important to seasonal predators [3–6]. In response to these types of environmental variability, migratory species may have to modify their spatio-temporal distribution and movement patterns, but the extent to which they can do so successfully is unclear [6–8].

Marine predators are generally experts in locating resources in patchy and dynamic marine environments, so they might be able to respond to interannually changing prey distributions [9,10]. However, animals undertaking long-distance migrations rely on learned information to inform their movements and time it to match resource peaks. Baleen whales, for example, show strong culturally transmitted philopatry to foraging and breeding grounds [11] and probably base their movements on memory of past resource distributions [1]. Both humpback whales (*Megaptera novaeangliae*) and fin whales (*Balaenoptera physalus*) have changed the timing of their migrations in response to earlier sea ice break up in the Gulf of St Lawrence over a 30 year period [12]. Additionally, changes in the migratory timing of humpback, blue (*Balaenoptera musculus*) and grey (*Eschrichtius robustus*) whales off California have been hypothesized to be driven by local oceanography, regional upwelling and basin-scale climate conditions [13]. Some recovering baleen whale populations are also re-populating historical foraging grounds decades after they had nearly been extirpated from over-exploitation [14,15].

When such changes in the phenology or distribution of migratory animals are observed, secondary effects on other parts of the annual cycle of migratory animals are expected [8,16]. However, these secondary effects are difficult to detect and may impact population vital rates, so it is important to consider them in context of the annual cycle to assess potential long-term effects [17]. Furthermore, dynamically changing spatio-temporal patterns of movement pose challenges to the management and monitoring of highly mobile animals [7]. It is therefore essential to describe the habitat use of migratory animals throughout the annual cycle and to integrate this knowledge into an ecosystem management framework [16]. This is particularly important where sensitive parts of a population, such as pregnant or nursing females, aggregate and in coastal regions where overlap with human activity is concentrated [18–20].

During the last decade, humpback whales in the North Atlantic have started to aggregate in fjord systems of northern Norway during the winters (between November and February), hereafter referred to as ‘northern Norway’ [21]. Here, they forage extensively on Norwegian spring-spawning (NSS) herring that shifted their wintering distribution into these areas [22–24]. This shift resulted in a dense and energy-rich prey resource along the migratory path of humpback whales [21,25]. NSS herring have shifted their wintering distribution regularly in the past [26], a phenomenon thought to be related to the stock’s age structure, potentially acting in conjunction with environmental changes [22,27]. Northeast Atlantic humpback whales generally forage throughout the Norwegian and Barents Seas during summer and autumn [28–30] and migrate to breeding grounds in the West Indies [31] and Cape Verde Islands [32], where most of them are observed in March–April and April–May, respectively. During the era of commercial whaling in the northeast Atlantic (1881–1904), humpback whales were caught off northern Norway in areas occupied by forage fish during the winter [30,33,34]. No substantial numbers of humpback whales have been observed in the fjords since then, especially not during wintertime, apart from occasional sightings of humpback whales by fishing and whale-watching vessels, which are common throughout Norwegian waters at most times of the year.

This novel or re-established foraging site appears to represent additional foraging opportunities for humpback whales after the presumed main summer foraging season, before their long southward migration towards tropical breeding grounds. Recent satellite tracking data and photographic matches have confirmed that animals observed during winter in northern Norway can still migrate to the breeding grounds during the same year [25,32]. However, no studies have quantified the connectivity between the Barents Sea and northern Norway, described the duration and spatial distribution of the foraging aggregation in northern Norway, or assessed whether the demographic composition in both feeding areas differs. The importance of northern Norway as a foraging opportunity for various demographic groups of humpback whales and the population should thus be explored in detail, given that the foraging season in northern Norway occurs unusually late in the year compared to the foraging seasons of humpback whales elsewhere.

In this study, we aimed to describe the foraging aggregation within the context of the northeast Atlantic humpback whales’ annual cycle, its demographic composition and spatio-temporal distribution. To this end, we used photographic identity (ID) matching to (i) quantify the connectivity between the Barents Sea and northern Norway, (ii) establish the duration and geographical distribution of the foraging area in northern Norway, and (iii) to assess the return rate of individual whales that foraged in the fjords of northern Norway both within and between years. Finally, we used genetic and hormone screening of biopsy samples to (iv) quantify the sex ratio and pregnancy rate of humpback whales in the Barents Sea and northern Norway.

## 2. Material and methods

### Study site and data collection

**Figure 1.**
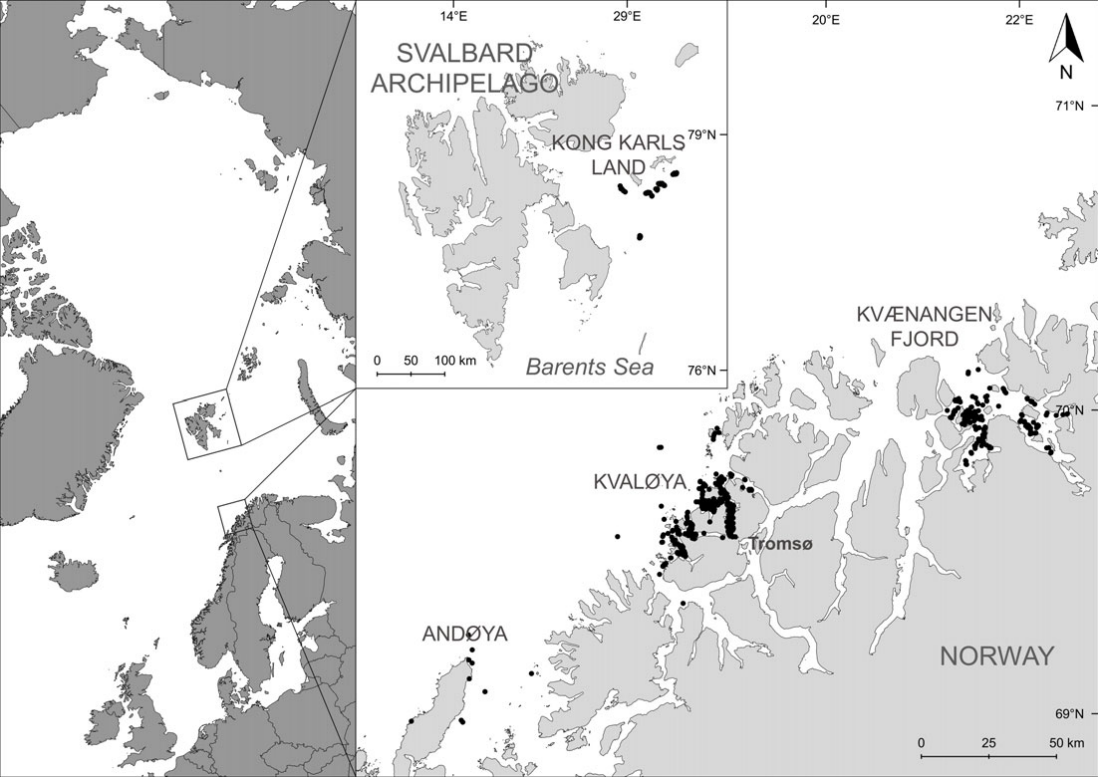
Left panel shows the Svalbard Archipelago with black dots close to Kong Karls Land representing GPS locations of photographic records of humpback whales (*Megaptera novaeangliae*). The inset shows the three main locations (Andøya, Kvaløya and Kvænangen fjord) of the northern Norwegian foraging area. Not all pictures were submitted with GPS locations, those without are not included in the figures.

We collected photo-identification data and biopsies in several fjords of northern Norway and waters of the Barents Sea surrounding the Svalbard Archipelago (figure 1). Northern Norway is not affected by sea ice during the winters, as it is characterized by warm north Atlantic water. The sea ice edge occurred around the Svalbard Archipelago during the peak of sea ice coverage in April between 2005 and 2018 (see [29]), and the area is generally free of ice between June and December. The North Norwegian Humpback Whale Catalogue (NNHWC) was established in 2010 when humpback whales started aggregating in northern Norway during the late autumn and winter. From hereon, we refer to ‘summer’ as the foraging season spanning June to September, and ‘winter’ as the foraging season from October to February. The study sites included waters around Andøya (2010–2012), Kvaløya (2012–2017) and Kvænangen (2017–2019) (figure 1). Photographic sampling was conducted using small vessels and was dictated by weather and light conditions. During the polar night (December–January), sampling was usually restricted to a few hours around midday. However, on some sampling trips, a flash system allowed sampling to continue in low-light conditions. The sampling effort differed between years and study sites (table 1). The public and other research organizations also submitted pictures, and an interactive online web portal for the submission of fluke photographs was established in 2015 (hvalid.no) and active until 2017, after which data collection continued with the existing network of contributors.

**Table 1.** Table of effort-based photo ID sampling and non-effort-based data collection for each location within the northern Norwegian fjords (2010/11 to 2018/19). (Sampling was mainly conducted by UiT and the founder of the NNHWC (effort-based). Other records (non-effort-based) represent days in which various contributors submitted fluke identification photographs. The period depicts the first and last humpback whale fluke capture in a season, with days indicating the duration between them, indicative of minimum season duration.)

	Andøya		Kvaløya		Kvænangen			
winter season	survey effort (days)	other records (days)	survey effort (days)	other records (days)	survey effort (days)	other records (days)	period	days
2010/2011	2	1					27 Dec–19 Jan	23
2011/2012	1	3	2				06 Dec–29 Jan	54
2012/2013		15	19	25			03 Nov–11 Feb	100
2013/2014		33	22	35			07 Nov–06 Feb	91
2014/2015		14	39	44			28 Oct–15 Feb	110
2015/2016		12	29	51			29 Oct–24 Feb	118
2016/2017			18	27		5	23 Oct–24 Jan	93
2017/2018					10	5	10 Nov–13 Jan	64
2018/2019					28	26	26 Oct–28 Jan	94
total	3	78	129	182	38	36		747
mean	1.5	13	21.5	36.4	19	12		83

From the 3–11 September 2018, a research cruise was conducted in cooperation between the Institute of Marine Research (IMR, Bergen, Norway) and UiT—The Arctic University of Norway (UiT, Tromsø, Norway), surveying the northern Barents Sea, east of the Svalbard archipelago close to the island group of Kong Karls Land (figure 1). We chose the timing and area based on information on humpback whale occurrence from prior annual joint Norwegian/Russian ecosystem surveys in the Barents Sea and adjacent waters (IMR, Norway/PINRO, Russia). When humpback whales were sighted, a small boat was launched to allow closer approaches. We took fluke photographs from both the small boat and the larger research vessel using DSLR cameras. In addition to this cruise, photographs from incidental humpback whale encounters around Svalbard, and the Barents Sea were submitted by various contributors (2012–2019), mostly nature-tourism expedition vessels that typically spend multiple weeks around Svalbard and Franz Josef Land, and to a smaller extent research cruises not targeted at marine mammals).

We took biopsies from either the fluke or flank of each individual from small open boats (20–26 ft) using an airgun (ARTS launching system, LKARTS-Norway) to deploy a floating arrow with a 4 or 6 cm long sterile stainless steel biopsy tip (CetaDart, DK). Depending on the shooting distance, usually about 4–20 m, the shooting pressure was between 6 and 10 bars.

Sampling procedures were approved by the Norwegian Food Safety Authorities (Mattilsynet), under permits FOTS-ID 14 135 and FOTS-ID 8165. We collected skin (*n* = 169) and blubber samples (*n* = 112) from humpback whales between 2011 and 2019 in the Troms area of northern Norway, and during September 2018 in the northern Barents Sea. Samples were stored at −20°C in either tin foil or glass vials (blubber) or 96% ethanol (skin).

### 2.1. Photo-identification

We identified individual humpback whales using the unique pigmentation pattern on their ventral flukes [35] and created sighting histories from re-identifications of photo-identified whales. Intervals between an individual’s first and last sightings within a season indicate the minimum length of stay during the season. We calculated the annual return rate, a measure of site fidelity on a population level, as the number of photographically recaptured individuals in a given year divided by the total number of individuals sighted in that year [36].

Individual sighting histories for this study relied on 3677 sightings of 1169 unique humpback whales documented in the NNHWC between 2010 and 2019. The catalogue covers a latitudinal range from 67° to $80^{\circ }\;N$. It contains sighting records of individual humpback whales throughout the year, with summer sightings mainly from the Barents Sea and winter sightings from northern Norway (figure 1). In northern Norway, we collected fluke photographs of 866 individual humpback whales, 856 (98.9%) of these during the winter. Most (54.7%) photographs were collected during dedicated sampling conducted between October and February, while remaining photos were contributed by third parties, including all summer sightings (1%).

Over 9 years of study, we conducted 170 days of dedicated photo-identification survey effort, with considerably less effort during the first two winters (table 1). The average annual sampling effort across all winter seasons was 17 days (±13.1) and 23.6 days (±9.4), excluding the first two seasons. We identified 342 individual whales in the Barents Sea, with most identification photographs (95%) obtained during a research cruise in September 2018. Other collaborators submitted fluke photographs from incidental humpback whale encounters between 2012 and 2019.

### 2.2. Sex determination

We determined the sex of individuals using skin samples [37], using the odontocete oligonucleotide primer set, ZFYX0582F, ZFY0767R and ZFX0923R, which showed clear bands on the gel electrophoresis. As a control, samples from four killer whales (*Orcinus orca*) of known sex (two males and two females) were used in every polymerase chain reaction. After initial testing, primer concentrations were optimized to 1 μl of 10 μM for the Y primer-set (ZFYX0582F/ZFY0767R) and 0.5 μl of 5 μM for the X primer-set (ZFYX0582F/ZFX0923R).

### 2.3. Resampling rate in biopsy material

To estimate the within-season recapture rate in our dataset, we conducted a relatedness analysis on a subset of the samples for which genetic sequences were available (*n* = 107). We used NGSrelate v2 [38] to calculate the coefficients of relatedness, based on genotype likelihoods calculated with ANGSD v.v0.935-53-gf475f10 [39]. See the electronic supplementary material, S2 text for more details.

### 2.4. Progesterone concentrations and pregnancy status

We used progesterone concentrations as a proxy for pregnancy status and extracted the progesterone from blubber samples as described in [40,41], with minor adjustments to the method. See the electronic supplementary material, S1 text and table S3 for more details. Progesterone was measured in 82 female blubber samples and 19 male control samples. Blubber samples taken from flukes were excluded since they usually do not contain enough blubber to conduct the analysis and may have different fat and hormone profiles leading to potential misclassifications.

We quantified progesterone concentrations using two commercially available progesterone enzyme immunoassays (EIA; Enzo Life Sciences, kit ADI-900-011 and ELISA; DRG International Inc. EIA-1561), see the electronic supplementary material, S1 text and table S3 for more details on the difference between the two methods. The dried hormone extract was re-suspended in 1 ml phosphate buffered saline (pH 7.5) containing 1% bovine serum albumin, vortexed, and then samples kept at −20°C. The EIA and ELISA kits we used have 100% reactivity with progesterone; the detection limit is between 15−500 pg ml^−^^1^ and 0−40 ng ml^−^^1^, respectively, based on the standard curves. Two additional standard dilutions were added to lower the detection limit of the EIA standard curve to 3.81 pg ml^−^^1^. We ran samples blind and in duplicate and re-ran samples that fell outside the detection limit at varying dilutions. The progesterone EIA’s inter-assay coefficient of variation (COV) and intra-assay COV ranged from 2.7–8.3% and 4.9–7.6%, respectively. The mean inter-assay COV was 14.7% for the EIA, and the mean intra-assay COV was 5.2% for the ELISA. Progesterone values are reported as nanograms per gram of blubber (ng g^−1^). We repeated the extraction and measurements for a subset of the blubber samples, in which case we report the averaged resulting progesterone level and ran multiple samples at several dilutions.

We assigned pregnancy status based on blubber progesterone concentrations using previously established models developed from female humpback whales of known pregnancy status from the Gulf of Maine and the Gulf of St Lawrence [40,41]. Previous studies successfully applied this modelling approach to other populations (e.g. western Antarctic Peninsula [41], Oceania [42]). Pregnancy rates were determined as the number of pregnant females divided by the total number of assayed females for years in which at least five samples were available, i.e. in which sample size allowed for reasonably robust estimation.

### 2.5. Statistical analysis

We checked whether the sex ratio deviated significantly from parity (1 : 1) for each region (northern Norway in winter, Barents Sea in summer) using a two-tailed exact binomial test for the Barents Sea, and one-tailed test for Norway. We then tested whether the pregnancy rate differed between the summer (samples obtained in June and September) and winter season (samples obtained between October and February in northern Norway), using a *χ*^2^ test of independence. Quasi-binomial generalized linear models (GLMs) were used to investigate variation in annual pregnancy rates between 2011 and 2018, and over the feeding season between June and February, using a ‘logit’ link function to take into account overdispersion in the pregnancy rate data. Given the limited and variable biopsy sample sizes and the variability in pregnancy rate estimates, it was important to consider these data in the context of their power to detect significant changes over time. The power of the GLMs was estimated using the pwr.f2.test function in the pwr package (R v. 3.6.2 [43]). The power to detect a trend in the pregnancy rate over the 8 year study period was 17.4%, and the power to detect a trend through the feeding season was 6.08%. Thus, the variability in pregnancy rate estimates makes the detection of significant temporal trends unlikely. A significance threshold of *p* < 0.05 was used to determine significance in all statistical tests. Results are presented as mean ± standard deviation, unless otherwise noted.

## 3. Results

### 3.1. Photographic collections

In northern Norway, the total number of photo-identified humpback whales per winter season ranged from a minimum of six individuals in the first year off Andøya (2010) to a maximum of 408 individuals in the 2015/2016 season off Kvaløya (figure 3; electronic supplementary material, table S1). The peak in sightings occurred between November and January. The cumulative curve of identifications began to plateau after the winter of 2015/2016 but showed a slight increase in 2018/2019 in Kvænangen (figure 3). In the Barents Sea, we registered humpback whale sightings from May to September, although most were photographed in September 2018. In total, we found five between-season re-sightings in the Barents Sea.

### 3.2. Connectivity between Barents Sea and Norway

**Figure 2.**
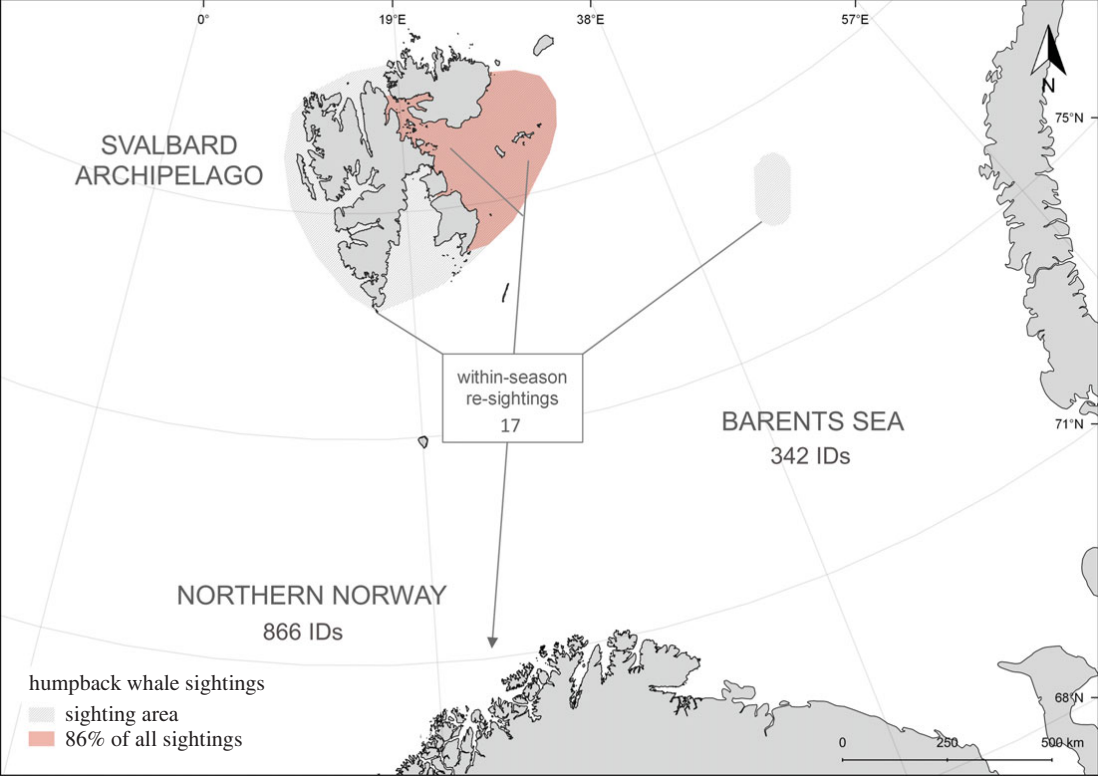
Map of the sampling area in the Barents Sea (in grey and orange) with the number of identified individuals in the Barents Sea (including the Svalbard Archipelago) and northern Norway, respectively, and the number of within-season matches between those two areas. The beginning of grey lines indicate first sighting locations within the Barents Sea of the individuals that were subsequently re-sighted in northern Norway. In total, 86% of all humpback whale IDs in the Barents Sea were collected in the orange-shaded area.

We matched 39 individual humpback whales sighted during summer in the Barents Sea to northern Norway during the winter (figure 2). One individual was photographed in two different summers in the Barents Sea and subsequently re-sighted off northern Norway during winter both these years. Seventeen matches of 16 individuals occurred within the same year (figure 2), showing that individuals transitioned between Barents Sea and northern Norway in the succession of one foraging season. Most of the re-sightings were first recorded in northern Norway at the end of November (electronic supplementary material, table S2).

### 3.3. Site fidelity in northern Norway

**Figure 3.**
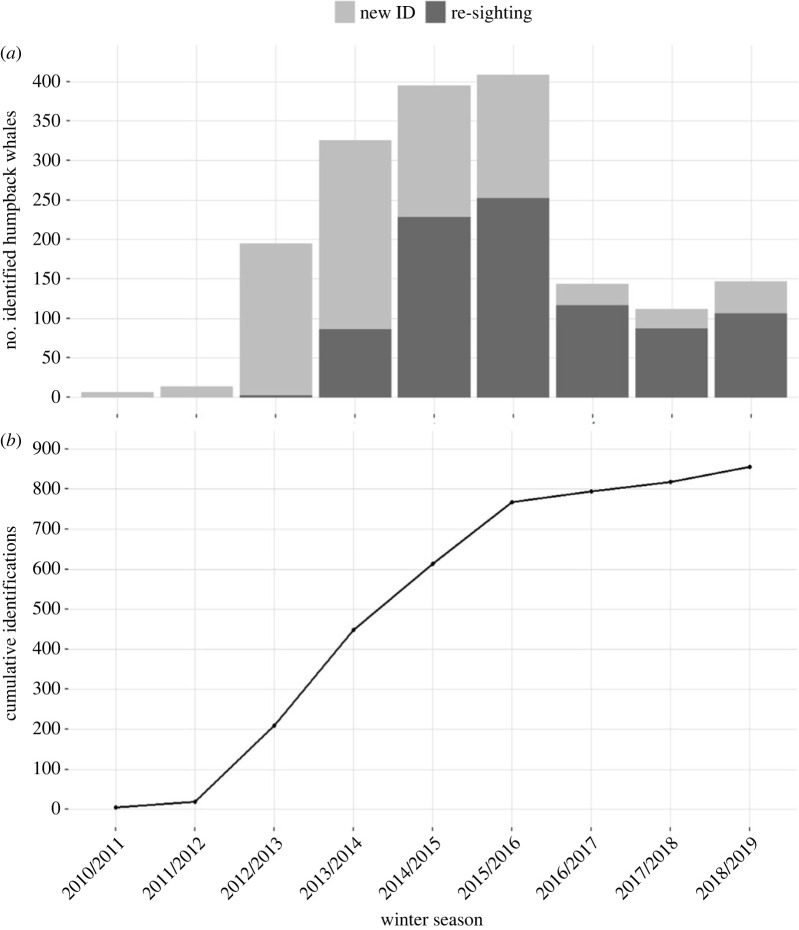
(*a*) Total number of individual humpback whales (*Megaptera novaeangliae*) photo-identified each winter season in northern Norway between 2010 and 2019. Light grey shading indicates newly identified individuals, and dark grey shading indicates re-sights of previously identified individuals. (*b*) Discovery curve illustrating the trend in the cumulative number of individual photo-identified humpback whales during winter in northern Norway (2010/2011 to 2018/2019).

**Figure 4.**
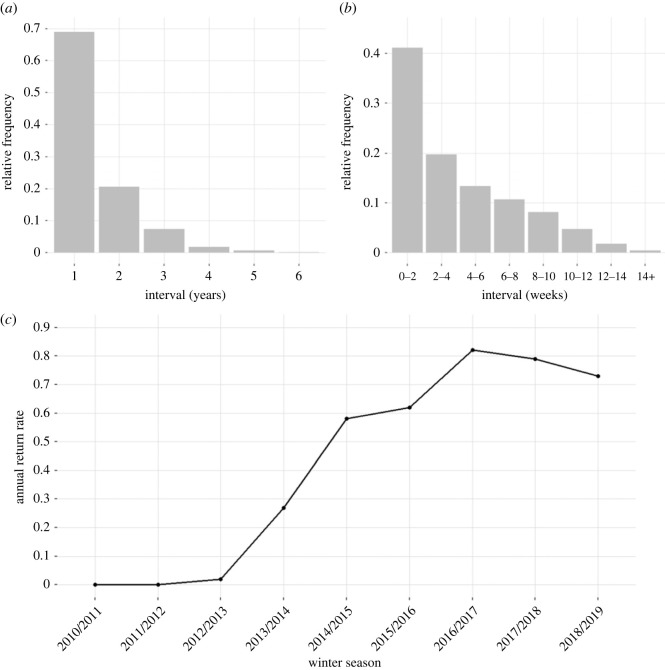
Upper panel: (*a*) Between season re-sighting intervals (*b*) within-season re-sighting intervals of humpback whales (*Megaptera novaeangliae*) in northern Norway (2012/13 to 2018/19). (*c*) Annual return rate of humpback whales to northern Norway (2010/2011 to 2018/2019).

Between the winter of 2010/2011 and 2018/2019, we photo-identified 866 individual humpback whales in northern Norway (figure 3). The majority (53.4%, *n* = 457) returned in two or more winters. Most of these whales were seen in two (*n* = 202), three (*n* = 131) or four (*n* = 83) different years. The longest period over which an individual was re-sighted was 7 years. Re-sightings between seasons occurred most frequently in sequential years (69.4%), followed by two-year intervals (20.6%) (figure 4). Until the winter of 2013/2014, new fluke captures accounted for more than 70% of the total number of whales identified in a season. In all following winters, the number of re-sightings was higher than first captures, on average 70.9% (±10.5) (figure 3).

The annual return rate, a measure of population-level site-fidelity, progressively increased until a peak in the 2016/2017 season (the final winter season off Kvaløya, 81.8%; figure 4), decreasing to 70 and 79% during the following two winter seasons (2017/2018, 2018/2019) in Kvænangen. Within a season 43.2% of the whales were seen more than once. The time interval between within-season re-sightings ranged from a minimum of 2 days to a maximum of 15 weeks, on average 27.5 days (±11.5; figure 3). More than half the whales identified across the 9 years of study were re-sighted, with 27% returning to feed for more than 3 years, most often in sequential years. In the winter of 2016/2017, considerably fewer humpback whales were encountered around Kvaløya, and the first individuals were sighted in Kvænangen fjord. In the consecutive winter, the fjords around Kvaløya were deserted, and the feeding activity had shifted to Kvænangen fjord.

### 3.4. Resampling rate in biopsy material

A relatedness analysis based on a subset of the samples for which genetic sequences were available (107 individuals) indicated that no individuals were biopsied repeatedly within the same season (coefficients of relatedness <1; electronic supplementary material, table S4).

### 3.5. Sex ratio

The sex ratio in the Barents Sea was 1.4 (18 M:13 F, *n* = 31) and in northern Norway 0.6 (48 M:76 F, *n* = 124). No significant deviation from parity was found for the Barents Sea sample (*p* = 0.473), but the sex ratio differed significantly from parity in northern Norway with a bias in favour of females (*p* = 0.007). The sex ratio in northern Norway differed significantly between years in our sample (*χ*^2^ = 12.9, *p* = 0.019). In years with low sample sizes (2011/2012, 2017/2018), the ratio of males in the sample was higher. The sex ratio did not differ significantly between months throughout the winter season (*χ*^2^ = 3.2, *p* = 0.571; electronic supplementary material, figure S1).

### 3.6. Pregnancy rate

All but three of the females for which blubber samples were available (*n* = 82) were successfully assigned a reproductive status (i.e. pregnant or non-pregnant) by the reference model (with 99.9% confidence), and all male controls (*n* = 19) were correctly classified as non-pregnant.

All progesterone concentrations are reported in the electronic supplementary material, table S3. The pregnancy rate was low in the summer (22% northern Norway in June, 20% Barents Sea in September 2018) and higher (median = 38%, 25th quantile = 24%, 75th quantile = 49%) during winter in northern Norway when pooled over all years (table 2). However, the difference between the Barents Sea and northern Norway in 2018/2019 (20% versus 47%) was not statistically significant (*χ*^2^ = 2, *p* = 1). Rates in winter varied across years between 8 and 56% (table 2). During the winter season, the pregnancy rate declined after a peak in December (73%), to 26% in January and 17% in February (table 3). Owing to the limited sample size and high variance, the power to detect a relationship in the pregnancy rate over winters in the 8 year study period was low (17.4%), and over the months during the feeding season even lower (6.1%). Thus, the variability in pregnancy rate estimates makes the detection of significant temporal trends unlikely.

**Table 2.** Numbers of female humpback whales assessed for progesterone levels and pregnancy rates in the Barents Sea and northern Norway by area and season. (The pregnancy rate (pregnant females/all assayed females) is reported for months with at least five samples.)

area	season	females	pregnant	not pregnant	pregnancy rate (%)
northern Norway	June 2011	2	1	1	—
	June 2012	7	1	6	14
Barents Sea	September 2018	10	2	8	20
northern Norway	winter 2013/2014	7	2	5	29
	winter 2015/2016	12	1	11	8
	winter 2016/2017	9	5	4	56
	winter 2017/2018	2	0	2	—
	winter 2018/2019	30	14	16	47
total		79	26	53	

**Table 3.** Numbers of female humpback whales assessed for progesterone levels and pregnancy rates in the Barents Sea and northern Norway by area and month. (The pregnancy rate (pregnant females/all assayed females) is reported for months with at least five samples.)

area	month	females	pregnant	not pregnant	pregnancy rate (%)
northern Norway	June	9	2	7	22
Barents Sea	September	10	2	8	20
northern Norway	October	2	0	2	—
	November	14	6	8	43
	December	11	8	3	73
	January	27	7	20	26
	February	6	1	5	17
total		79	26	53	

## 4. Discussion

Within-season matches between the Barents Sea and northern Norway confirm that some northeast Atlantic humpback whales continued their foraging season in fjord systems of northern Norway. Studies on other humpback whale feeding grounds have shown that females generally leave feeding grounds later than males, resulting in a female bias late in the foraging season [11,41,44] consistent with our observation of a female bias in northern Norway but not the Barents Sea. Per our expectations, the pregnancy rate estimated during winter in northern Norway was higher than in June and September, indicating that pregnant animals may indeed be more likely than the general population to maximize their energy intake by continuing their foraging season in northern Norway. An increase in pregnancy rates in the temporal progression of the foraging season was also observed in other areas ([41]; summer 59%, autumn 72%), a pattern consistent with knowledge obtained from whaling data [44].

The establishment of the foraging site in northern Norway coincided with dense herring concentrations in the area since 2010 (documented in detail since 2015 by [22]). During the overwintering period between October and February, the NSS-herring spawning stock can use separate areas concurrently including near and offshore waters [27], and fishing vessels reported that individual humpback whales foraged further offshore in previous years. Before 2010, the overwintering distribution of this herring stock was concentrated in fjords further south, and humpback whales were not present at this site [21]. Shifts in NSS-herring overwintering distribution have occurred repeatedly and are most likely related to changes in the stock’s age structure mediated by oceanographic conditions and fishing pressure [26,27]. Since humpback whales established the northern Norway winter foraging aggregation in 2010, NSS-herring slightly shifted their distribution northwards within the fjord systems until 2019, followed by a corresponding shift in whale distribution. The high annual return rate, comparable to main feeding grounds in other areas [36,45,46], indicates that foraging in northern Norway has become an important part of the annual routine for some northeast Atlantic humpback whales. Since the feeding activity is coupled to herring overwintering distribution, future shifts in the whales’ winter distribution can be expected as the herring stock changes its migration patterns and overwintering areas.

Information on the migration phenology of northeast Atlantic humpback whales remains sparse owing to the logistic challenges involved in surveying the Barents Sea region. Therefore, the duration of the summer foraging season is unknown. Our sighting data from the Barents Sea confirm that the area east of the Svalbard archipelago is an important foraging ground for humpback whales in late summer/autumn. This supports previous evidence from annual ecosystem surveys, whaling records and tracking data [29,47,48]. Tracking data from 2018 indicates that whales initiated migration from the Barents Sea between October and December in 2018, the same year most sightings and all biopsies were collected in the Barents Sea [29].

Within-season resighting patterns in northern Norway show that most whales stayed longer than two weeks, many for about one month and some up to three months. This should be considered a minimum estimate, as whales might arrive before their first sighting or stay after the last recorded sighting. In the north Pacific, groups of humpback whales have also been observed foraging on herring during some periods of the winter, however, this seems to be representing smaller numbers of whales than in the present study [49,50]. In Iceland, humpback whales have also been reported throughout the year [51]. The humpback whale aggregation in northern Norway is to our knowledge the largest, longest-lasting, and most stable documented winter foraging aggregation.

Photographic matching to the breeding grounds in the West Indies [31], along with a recently recorded round-trip migration by a female humpback whale [25] and unpublished tracking data show that many animals migrate to breeding areas after foraging in northern Norway during the winter. However, pregnant females delaying their migration until late in the season may give birth along the migration route despite increasing their migration speed [25], indicating carry-over effects from the long foraging season into the next stages of migration.

Our results provide, to our knowledge, a first indication that pregnant females might preferentially visit northern Norway as a continuation of the feeding season in the Barents Sea. When we restricted the analysis to the one year for which we had sampled the Barents Sea and northern Norway, small sample sizes however meant that our analysis lacked statistical power to conclude this with certainty. We could not confirm that pregnant females remained the longest in northern Norway. However, the statistical power to detect temporal trends in our data was low. One explanation for the lower pregnancy rates at the end of the season (January/February) may be that not all humpback whales complete migrations every year. Juvenile individuals and resting females for whom the cost and risks outweigh the benefits of migration may therefore dominate the sample towards the end of the season. This might contribute to lower pregnancy rates among females sampled, as well as an increase in the proportion of males in February.

Monitoring pregnancy rates over time can indicate population health and growth rates, provided that the sample sizes are sufficient [40,41]. Our estimate of the variation in pregnancy rate between years is probably not sufficiently robust to infer trends in reproductive rates, owing to the low number of samples in some years. Overall, the pregnancy rate in summer and winter was lower than those reported on other foraging grounds. On other humpback whale feeding grounds, pregnancy rates were reported to be higher, for example, 57% in the Southern Ocean [42], 58% (36–86%) in the western Antarctic Peninsula [41], 19–48% in the north Pacific [52] and 25–63% in the northwest Atlantic [40]. Previous pregnancy rate assessments in north Atlantic humpback whales did not detect an increase in blubber progesterone concentrations between females sampled early and late in the season ([24,40]), so this is probably not the sole driver of the increase. The variability between years reported here was similarly high in those other studies. Pregnancy rate estimates present a minimum of true rates, as they usually include immature females. Pregnancy rates sampled at different times of the gestation period may vary, e.g. be inflated by subsequently aborted/reabsorbed pregnancies when sampled early [41,42]. However, the large effect size of the difference in pregnancy rates during summer versus winter in our results was indicative of a true difference. Recent work shows that pregnancy rates are tightly linked to fluctuations in prey availability in the Antarctic, north Pacific and north Atlantic [5,40,53,54]. Further studies should assess whether low pregnancy rates here may indicate slowing population growth following recovery from exploitation [28] and resulting population density effects in a recovering population of northeast Atlantic humpback whales foraging in the Barents Sea, or poor nutritional status owing to changing environmental conditions and prey availability as is the case for humpback whales in other areas [5,53,54].

Rapid and fundamental ecosystem changes in the Barents Sea associated with warming, sea ice loss and increased inflow of Atlantic waters have impacted a core foraging habitat of humpback whales [29,55]. Further south in the Norwegian Sea, sightings of humpback whales have been less common during summers in 2009–2012 in contrast to the years 2006–2007, indicating a northward shift of foraging activity or changing migration timing on their northward journeys [48]. In general, humpback whale populations have shown remarkable recovery after historical exploitation [41,54,56,57]. Yet, their reproductive success is tightly coupled to prey availability [5], and humpback whale populations in the northwest Atlantic and north Pacific have been experiencing declining calving rates, probably owing to ecosystem shifts mediated by climate change [40,54].

In the case of northeast Atlantic humpback whales, herring superabundance events inside fjord systems provided a feeding opportunity outside of the presumed core feeding season, but along whales’ distributional range or migratory paths. The recent shift of herring distribution may have made this resource more accessible to whales since it now occurs closer to migratory routes [25] and might be more densely aggregated in fjord systems, in contrast to wintering areas herring occupied during the last decades [22,27]. Northern Norway could be considered a spatial continuation of the foraging area in the Barents Sea, potentially extending the duration of the foraging season, or a stopover after the commencement of southward migration from the Barents Sea. As northeast Atlantic humpback whales recover to historical abundance [28], density-dependent resource competition in the Barents Sea might play a role in changed distribution patterns. Increased whale abundances, potentially in conjunction with ecosystem changes, might lead to increased resource competition and more exploratory foraging movements outside of the main foraging areas.

As generalist predators, humpback whales are thought to be adaptable to changes in their prey distribution and abundance, relative to other baleen whale species. However, they certainly will not be able to respond to all kinds of changes in the structure of prey fields, as has been documented in other areas [5,40,53]. It is further unclear how late-season foraging, as documented in this study, affects the annual cycle of northeast Atlantic humpback whales. Since migratory species rely on habitats that are spread over vast distances and multiple jurisdictions, managing these habitats becomes an international responsibility. Dynamic management of ocean and coastal ecosystems that can account for changes in spatio-temporal distributions is a challenging but necessary task for the future that requires concerted efforts from multiple actors and potential protection during migration in areas beyond national jurisdiction [7]. Climate change is projected to severely impact population vital rates and alter distributions of top predators on longer time scales [3,58–60]. Therefore, continued monitoring of the pregnancy or calving rate in this population is warranted as the ecosystems of the Barents and Norwegian seas shifts to a new ecological state [29,55]. Knowledge of year-round distributions and critical habitat, especially during potentially vulnerable periods such as pregnancy, are essential for mitigating adverse effects of human activities on top predators [7].

## 5. Conclusion

Our results suggest that winter foraging on fjord-based herring is a strategy that is preferentially used by female humpback whales in northern Norway. Our findings suggest that this strategy has become an important annual event for humpback whales, contingent on herring overwintering in these fjords. The population of humpback whales in the northeast Atlantic is recovering from historical exploitation, while the ecosystem in which they forage is undergoing rapid changes. The establishment of this foraging site is evidence of humpback whales’ ability to respond flexibly to prey resources along their migratory pathways, with potential effects on their migration timing that need further investigation. Monitoring the potential anthropogenic impacts on migratory species as their distributions respond to changing environmental conditions, with special attention to core foraging areas, will be important to ensure adverse impacts can be recognized and addressed. In particular, if many of the whales visiting northern Norway during winter are either pregnant or are part of the endangered population segment that migrates to the Cape Verde breeding ground, potential impacts of the shipping and fishing industries should be priorities for ecosystem management. Future work should also aim to understand how this additional foraging opportunity impacts the overall reproductive performance and annual schedules of individual whales, and how this ultimately may affect population dynamics.

## Data Availability

The data are provided in the electronic supplementary material [61].
